# Supplementary material for: Perturbation of Copper Homeostasis Sensitizes Cancer Cells to Elevated Temperature
Source: Int J Mol Sci. 2023 Dec 28;25(1):423. doi: 10.3390/ijms25010423 (PMC10779418; doi:10.3390/ijms25010423)
Supplement: Supplementary file 1 [file ijms-25-00423-s001.zip › FigureS4.pdf]

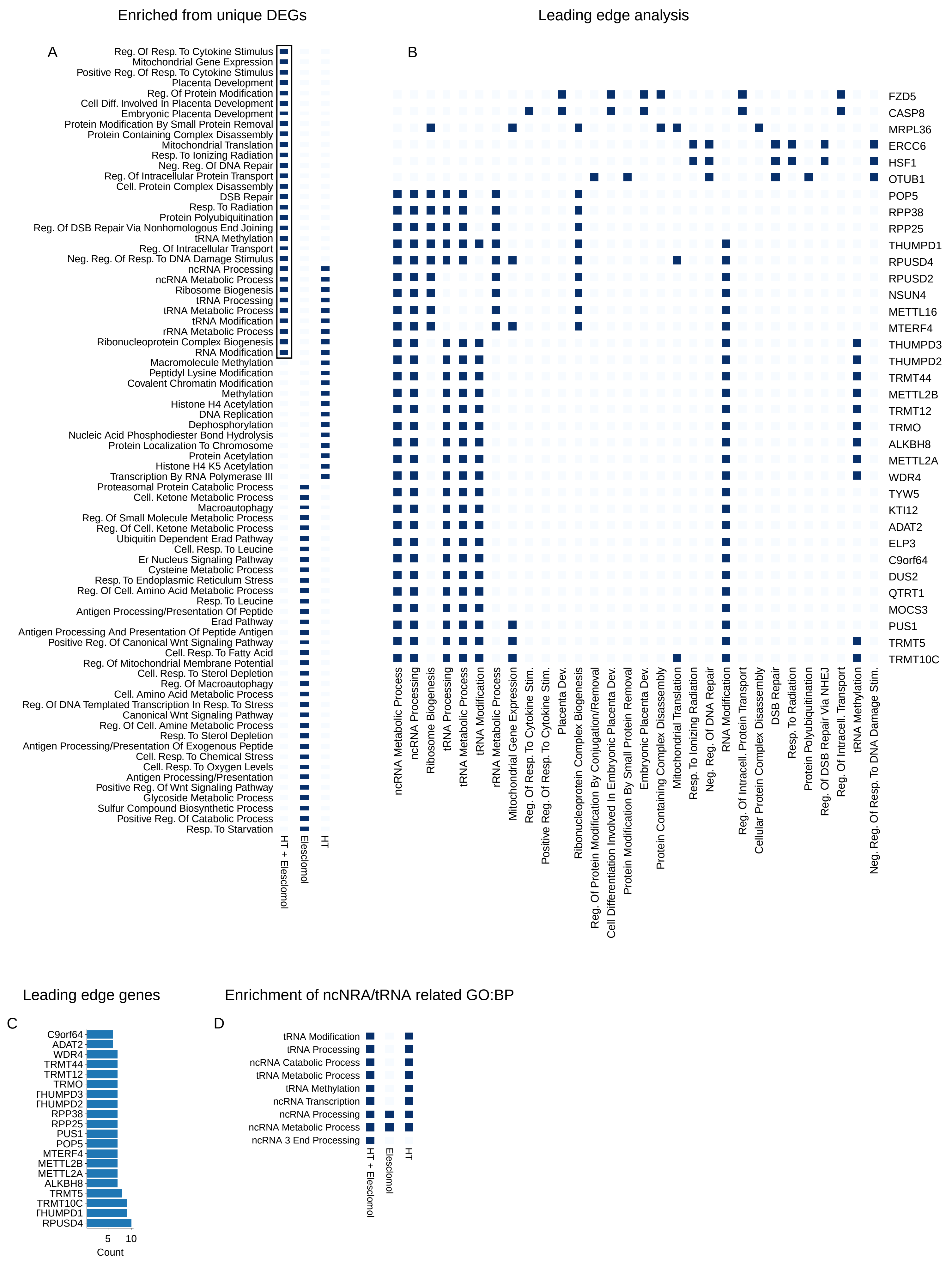
Enriched from unique DEGs
Leading edge analysis
A
Reg. Of Resp. To Cytokine Stimulus
Mitochondrial Gene Expression
Positive Reg. Of Resp. To Cytokine Stimulus
Placenta Development
Reg. Of Protein Modification
Cell Diff. Involved In Placenta Development
Embryonic Placenta Development
Protein Modification By Small Protein Removal
Protein Containing Complex Disassembly
Mitochondrial Translation
Resp. To Ionizing Radiation
Neg. Reg. Of DNA Repair
Reg. Of Intracellular Protein Transport
Cell. Protein Complex Disassembly
DSB Repair
Resp. To Radiation
Protein Polyubiquitination
Reg. Of DSB Repair Via Nonhomologous End Joining
tRNA Methylation
Reg. Of Intracellular Transport
Neg. Reg. Of Resp. To DNA Damage Stimulus
ncRNA Processing
ncRNA Metabolic Process
Ribosome Biogenesis
tRNA Processing
tRNA Metabolic Process
tRNA Modification
rRNA Metabolic Process
Ribonucleoprotein Complex Biogenesis
RNA Modification
Macromolecule Methylation
Peptidyl Lysine Modification
Covalent Chromatin Modification
Methylation
Histone H4 Acetylation
DNA Replication
Dephosphorylation
Nucleic Acid Phosphodiester Bond Hydrolysis
Protein Localization To Chromosome
Protein Acetylation
Histone H4 K5 Acetylation
Transcription By RNA Polymerase III
Proteasomal Protein Catabolic Process
Cell. Ketone Metabolic Process
Macroautophagy
Reg. Of Small Molecule Metabolic Process
Reg. Of Cell. Ketone Metabolic Process
Ubiquitin Dependent Erad Pathway
Cell. Resp. To Leucine
Er Nucleus Signaling Pathway
Cysteine Metabolic Process
Resp. To Endoplasmic Reticulum Stress
Reg. Of Cell. Amino Acid Metabolic Process
Resp. To Leucine
Antigen Processing/Presentation Of Peptide
Erad Pathway
Antigen Processing And Presentation Of Peptide Antigen
Positive Reg. Of Canonical Wnt Signaling Pathway
Cell. Resp. To Fatty Acid
Reg. Of Mitochondrial Membrane Potential
Cell. Resp. To Sterol Depletion
Reg. Of Macroautophagy
Cell. Amino Acid Metabolic Process
Reg. Of DNA Templated Transcription In Resp. To Stress
Canonical Wnt Signaling Pathway
Reg. Of Cell. Amine Metabolic Process
Resp. To Sterol Depletion
Antigen Processing/Presentation Of Exogenous Peptide
Cell. Resp. To Chemical Stress
Cell. Resp. To Oxygen Levels
Antigen Processing/Presentation
Positive Reg. Of Wnt Signaling Pathway
Glycoside Metabolic Process
Sulfur Compound Biosynthetic Process
Positive Reg. Of Catabolic Process
Resp. To Starvation
HT + Elesclomol
Elesclomol
HT
B
FZD5
CASP8
MRPL36
ERCC6
HSF1
OTUB1
POP5
RPP38
RPP25
THUMPD1
RPUSD4
RPUSD2
NSUN4
METTL16
MTERF4
THUMPD3
THUMPD2
TRMT44
METTL2B
TRMT12
TRMO
ALKBH8
METTL2A
WDR4
TYW5
KTI12
ADAT2
ELP3
C9orf64
DUS2
QTRT1
MOCS3
PUS1
TRMT5
TRMT10C
ncRNA Metabolic Process
ncRNA Processing
Ribosome Biogenesis
tRNA Processing
tRNA Metabolic Process
tRNA Modification
rRNA Metabolic Process
Mitochondrial Gene Expression
Reg. Of Resp. To Cytokine Stim.
Positive Reg. Of Resp. To Cytokine Stim.
Placenta Dev.
Ribonucleoprotein Complex Biogenesis
Reg. Of Protein Modification By Conjugation/Removal
Cell Differentiation Involved In Embryonic Placenta Dev.
Protein Modification By Small Protein Removal
Embryonic Placenta Dev.
Protein Containing Complex Disassembly
Mitochondrial Translation
Resp. To Ionizing Radiation
Neg. Reg. Of DNA Repair
RNA Modification
Reg. Of Intracell. Protein Transport
Cellular Protein Complex Disassembly
DSB Repair
Resp. To Radiation
Protein Polyubiquitination
Reg. Of DSB Repair Via NHEJ
Reg. Of Intracell. Transport
tRNA Methylation
Neg. Reg. Of Resp. To DNA Damage Stim.
Leading edge genes
Enrichment of ncNRA/tRNA related GO:BP
C
C9orf64
ADAT2
WDR4
TRMT44
TRMT12
TRMO
THUMPD3
THUMPD2
RPP38
RPP25
PUS1
POP5
MTERF4
METTL2B
METTL2A
ALKBH8
TRMT5
TRMT10C
THUMPD1
RPUSD4
5
10
Count
D
tRNA Modification
tRNA Processing
ncRNA Catabolic Process
tRNA Metabolic Process
tRNA Methylation
ncRNA Transcription
ncRNA Processing
ncRNA Metabolic Process
ncRNA 3 End Processing
HT + Elesclomol
Elesclomol
HT
