# Supplementary material for: Perturbation of Copper Homeostasis Sensitizes Cancer Cells to Elevated Temperature
Source: Int J Mol Sci. 2023 Dec 28;25(1):423. doi: 10.3390/ijms25010423 (PMC10779418; doi:10.3390/ijms25010423)
Supplement: Supplementary file 1 [file ijms-25-00423-s001.zip › ijms-2762882-supplementary.pdf]

Supplementary Materials

Supplementary figure legends

Figure S1. Discovery of novel thermosensitizing agents. (**A**) Sensitivity of cells to the ATP synthase inhibitor FCCP in glucose- or galactose-rich media (GLU/GAL), which reduces (GLU) or and enhances (GAL) the dependency of cells on mitochondrial energy production. (**B**) Cell viability 48 hours after HT in glucose- or galactose-rich media. (**C**) Thermal enhancement ratio (TER) of mono- and combination therapy with inhibitors of glutamine oxidation (BPTES), fatty acid oxidation (etoxomir), and pyruvate oxidation (UK-5099), derived from cell viability assays performed 72 hours after hyperthermia (HT). (**D**) Cell viability 72 hours after exposure to combinations of HT, the ROS-inducer menadione, and the ROS scavenger NAC (2 mM). Asterisks mark significant differences between the non heated control and hyperthermia arm.

Figure S2. Comparison of the thermosensitizing effects of elesclomol in benign and malign cells. (**A**) Cell viability of benign and bladder cancer cell lines, 72 hours after exposure to elesclomol and/or hyperthermia (HT). (**B**) Inhibitory concentration of elesclomol that reduces cell viability by 50% (IC50) in the presence or absence of HT. (**C**) IC50-based thermal enhancement ratio (TER), derived from (**B**). Asterisks mark significant differences.

Figure S3. Effects of combined treatment with elesclomol and hyperthermia on proteostasis. (**A**) Immuno-blot showing HSP70i and beta-actin (loading control) expression, 6 hours after treatment with hyperthermia (HT) and/or elesclomol (1 µM). (**B**) Quantification of HSP70i expression in (**A**), normalized to beta-actin. Asterisks mark significant differences in relation to the non heated control. (**C**) Immuno-blot showing ubiquitin (Ub) and beta-actin (loading

control), 0.5 and 6 hours after HT. Elesclomol was used at a concentration of 1 µM. (**D**) Quantification of total Ub expression in (**C**), normalized to beta-actin.

Figure S4. Transcriptomic alterations caused by combined treatment with elesclomol and hyperthermia. Genes that were uniquely differentially expressed in each condition, as shown in Figure 5E, were used as input for a gene set enrichment analysis of Gene Ontology (GO) Biological Processes. (**A**) Significantly enriched GO terms (dark blue) after treatment with hyperthermia (HT), elesclomol, or combined. (**B**) Leading edge analysis of GO terms that were found significantly enriched after combined treatment with elesclomol and hyperthermia. (**C**) Ranked list of genes that contributed mostly to the leading edge. Several genes were found to drive the enrichment of GO terms related to non-coding RNA (ncRNA) or transfer RNA (tRNA). (**D**) We re-checked the enrichment of GO terms per experimental condition (Figure 5F) to evaluate whether ncRNA or tRNA-related processes are uniquely differentially regulated after combined treatment. The alteration of these processes appears to be shared, however, between the combination therapy and hyperthermia monotherapy.

Figure S5. Assessing the role of ROS induction and oxidative phosphorylation in the thermosensitizing phenotype of elesclomol. (**A**) Heatmap showing normalized survival directly after treatment with elesclomol (1 µM), hyperthermia (HT), or the combination. (**B**) Pearson correlation between survival shown in (**A**) and ATP levels. (**C**) Superoxide levels after treatment with electron transport chain complex III inhibitor Antimycin A (50 µM), in the presence or absence of the superoxide scavenger MitoQ (0.5 µM). Superoxide caused by Antimycin A can be compensated for by MitoQ. Serves as a positive control for figure 5D. (**D**) Mitochondrial membrane potential (MMP) directly after a one hour exposure to ATP synthase inhibitor FCCP (100 µM). MMP is effectively decreased after FCCP treatment. Serves as a positive control for figure 5A. (**E**) Total ROS levels in three cell lines one hour after exposure

to the ROS inducer menadione and/or ROS scavenger NAC (2 mM). Total ROS levels are lowered in the presence of NAC. Serves as a positive control for figure 5C. (**F**) Cell viability 72 hours after exposure to combinations of HT and elesclomol in the presence or absence of ferroptosis inhibitor ferrostatin-1 (15 μM). Asterisks mark significant differences.

Figure S6. Cuproptosis does not drive thermosensitization by elesclomol. (**A**) Viability of wildtype and FDX1-2 KO cells, 72 hours after a 1 or 2-hour pulsed treatment with elesclomol. Serves as a positive control for figure 6D.
