# Supplementary figures and images for: Perturbation of Copper Homeostasis Sensitizes Cancer Cells to Elevated Temperature
Source: Int J Mol Sci. 2023 Dec 28;25(1):423. doi: 10.3390/ijms25010423 (PMC10779418; doi:10.3390/ijms25010423)

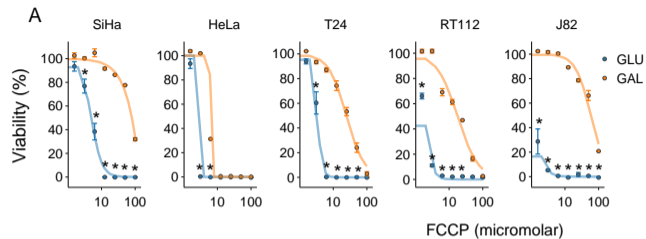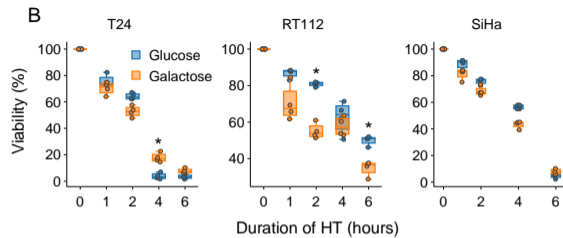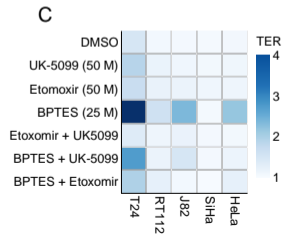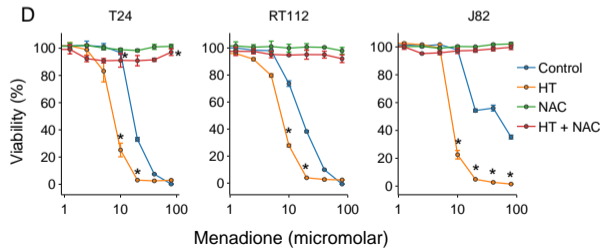

Supplement: Supplementary file 1 [file ijms-25-00423-s001.zip › FigureS1.pdf]

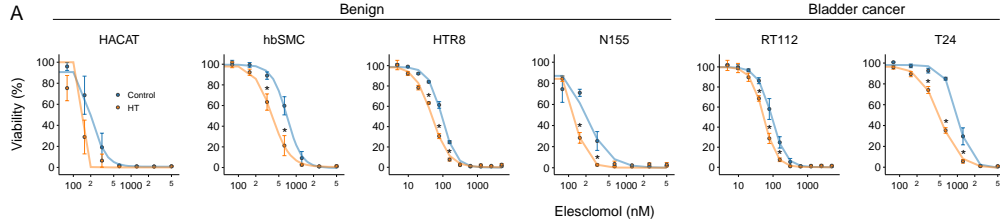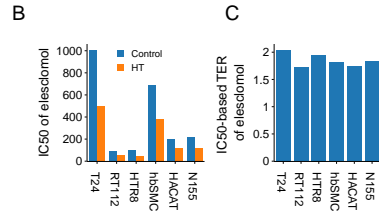

Supplement: Supplementary file 1 [file ijms-25-00423-s001.zip › FigureS2.pdf]

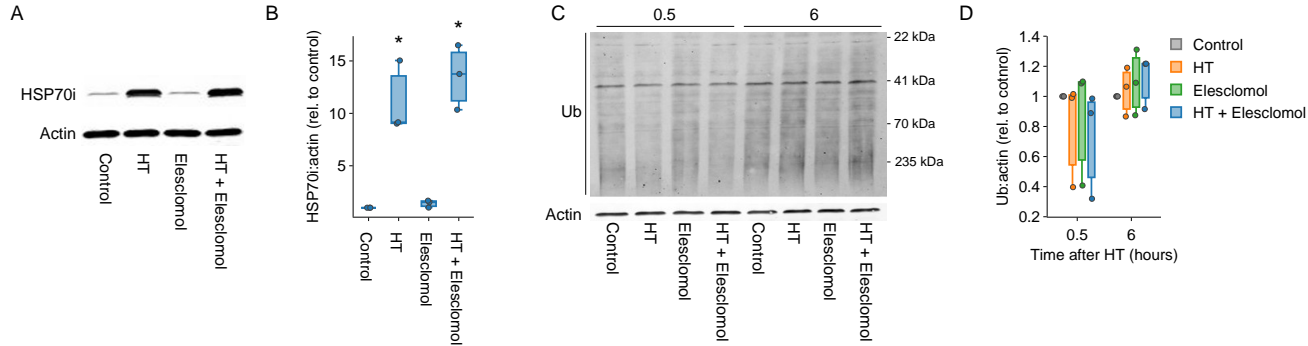

Supplement: Supplementary file 1 [file ijms-25-00423-s001.zip › FigureS3.pdf]

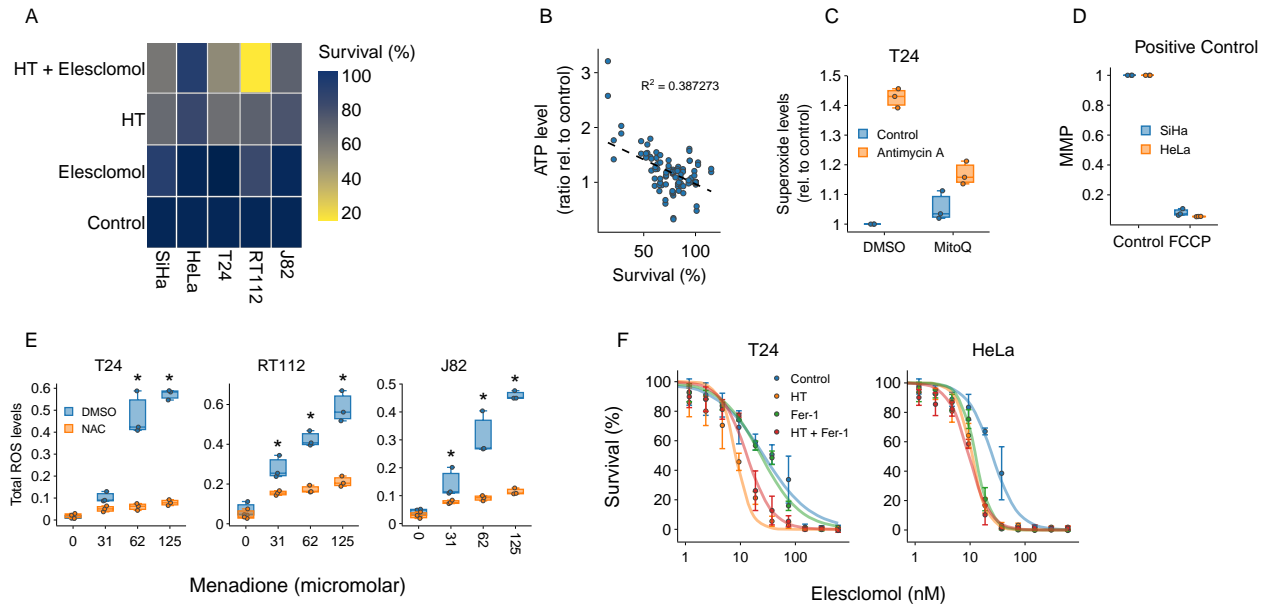

Supplement: Supplementary file 1 [file ijms-25-00423-s001.zip › FigureS5.pdf]

A

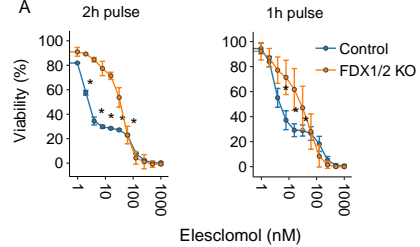

Supplement: Supplementary file 1 [file ijms-25-00423-s001.zip › FigureS6.pdf]
